# Improvement in serum eosinophilia is observed in clinical responders to ustekinumab but not adalimumab in inflammatory bowel disease

**DOI:** 10.1093/ecco-jcc/jjaf006

**Published:** 2025-01-13

**Authors:** Emily C L Wong, Parambir S Dulai, John K Marshall, Vipul Jairath, Walter Reinisch, Neeraj Narula

**Affiliations:** Department of Medicine (Division of Gastroenterology) and Farncombe Family Digestive Health Research Institute, McMaster University, Hamilton, ON L8S 4K1, Canada; Division of Gastroenterology, Northwestern University, Chicago, IL 60208, United States; Department of Medicine (Division of Gastroenterology) and Farncombe Family Digestive Health Research Institute, McMaster University, Hamilton, ON L8S 4K1, Canada; Department of Medicine, Division of Gastroenterology, Western University, London, ON N6A 3K7, Canada; Department of Internal Medicine III, Division of Gastroenterology and Hepatology, Medical University of Vienna, Währinger Gürtel 18-20, Vienna 1090, Austria; Department of Medicine (Division of Gastroenterology) and Farncombe Family Digestive Health Research Institute, McMaster University, Hamilton, ON L8S 4K1, Canada

**Keywords:** eosinophils, inflammatory bowel disease, ustekinumab, adalimumab

## Abstract

**Introduction:**

In inflammatory bowel disease (IBD), the number of eosinophils increases in the lamina propria of the intestinal tract, but their specific patho-mechanistic role remains unclear. Elevated blood eosinophil counts in active IBD suggest their potential as biomarkers for predicting response to biological therapies. This study evaluates blood eosinophil count trends and their predictive value for clinical response and endoscopic improvement in patients with IBD receiving ustekinumab or adalimumab induction therapy.

**Methods:**

Participant-level data from phase 3 and 4 clinical trials (UNIFI, SEAVUE, VARSITY) evaluating ustekinumab and adalimumab for moderate–severe Crohn’s disease (CD) and ulcerative colitis (UC) were used. The primary outcome was clinical response, defined by reductions in disease activity scores. Eosinophil counts were compared between responders and non-responders at multiple time points using *t*-tests. Logistic regression assessed the odds of achieving a clinical response based on baseline eosinophil counts.

**Results:**

Among patients treated with ustekinumab for UC, responders had significantly higher baseline eosinophil counts compared to non-responders (0.21 × 10^9^/L vs 0.18 × 10^9^/L, *P* = .042). By week 8, responders showed a greater absolute (−0.07 × 10^9^/L vs −0.01 × 10^9^/L, *P* < .001) and percent decline (−33.33% vs −5.55%, *P* = .027) in eosinophil counts. In CD, ustekinumab responders also had higher baseline eosinophil counts and showed significant reductions by week 8. However, no significant differences in eosinophil counts were observed among CD patients treated with adalimumab or UC patients treated with vedolizumab.

**Conclusion:**

Eosinophil reduction was identified as a marker for early response to ustekinumab in both UC and CD, but not adalimumab. No difference was observed among UC patients treated with vedolizumab either. Targeting the IL-12/IL-23 pathway may be more effective in managing eosinophil-associated inflammation in IBD.

## 1. Introduction

Inflammatory bowel disease (IBD), which includes ulcerative colitis (UC) and Crohn’s disease (CD), is a chronic immune-mediated disease that causes inflammation of the gastrointestinal tract.

Eosinophils, a type of white blood cell involved in immune responses, have been implicated in the pathophysiology of several inflammatory and autoimmune conditions, including UC and CD. Under normal conditions, eosinophils in the gastrointestinal tract reside within the lamina propria of the stomach and intestine. When eosinophils are recruited to intestinal tissue, eosinophils induce tissue damage through the secretion of several inflammatory mediators, including eosinophilic cationic protein, major basic protein, eosinophil protein X, eosinophil-derived neurotoxin, and eosinophil peroxidase.^1^

While the exact cause of IBD remains unknown, its pathogenesis likely involves an antigenic stimulus in conjunction with a genetic predisposition, resulting in the increased production of chemo-attractants that recruit inflammatory cells, such as eosinophils, to the gastrointestinal tract. Once recruited, eosinophils release toxic proteins and cytokines that may further contribute to the inflammatory process.^2,3^ Elevated levels of eotaxin and IL-5 have been observed in active IBD, correlating with higher blood eosinophil numbers and activation.^4^

Given their potential role in the pathogenesis of IBD, there is a growing area of interest in evaluating eosinophils as a biomarker for predicting responses to biological therapies. Ustekinumab, a monoclonal antibody targeting IL-12 and IL-23, and adalimumab, an anti-tumor necrosis factor (TNF) antibody, are commonly used biologics for treating UC and CD.^5^ Identifying markers of early response to these therapies is of paramount importance. Early identification of responders and non-responders can significantly impact treatment strategies, allowing for more personalized and effective management of IBD. This study aims to evaluate trends in eosinophil counts and their predictive value for clinical response and endoscopic improvement in patients undergoing induction therapy with ustekinumab or adalimumab.

## 2. Methods

### 2.1. Study design

This study included participant-level data from several phase 3 and 4 clinical trials (UNIFI, SEAVUE, and VARSITY) evaluating ustekinumab and adalimumab for the treatment of moderate–severe CD and UC. Data were obtained through Vivli protocol #00007656. Data from the UNIFI study (Clinicaltrials.gov: *NCT02407236*) were obtained with permission from Janssen Inc.^6^ Briefly, the UNIFI study assessed the efficacy and safety of ustekinumab among participants with moderate–severe UC. During the induction phase of the study, participants were randomly assigned to receive 8 weeks of ustekinumab intravenously as a fixed dose, weight-based dose, or placebo. Those with a clinical response by week 8 were re-randomized to receive subcutaneous ustekinumab every 12 weeks, every 8 weeks, or placebo for an additional 44 weeks. Data from the SEAVUE trial (Clinicaltrials.gov: *NCT03464136*) was also obtained with permission from Janssen Inc. for this analysis. Eligible participants included those who were biologic-naïve with moderate–severely active CD and evidence of endoscopic disease. Participants were randomly assigned to receive ustekinumab or adalimumab through week 56.^7^ Finally, data from the VARSITY trial (Clinicaltrials.gov: *NCT02497469*) were obtained by permission from Takeda Inc.^8^ Briefly, VARSITY was a head-to-head trial evaluating the efficacy and safety of vedolizumab and adalimumab in the treatment of moderate-severe UC. Patients were randomly assigned to receive vedolizumab or adalimumab at baseline, week 2, 6, and every 8 weeks thereafter to week 52. For this analysis, only participants who received adalimumab were included.

### 2.2. Outcomes

The primary outcome was to determine the predictive value of blood eosinophil counts for clinical response in patients receiving ustekinumab or adalimumab. For the ustekinumab UC analyses, clinical response was defined as a reduction in total Mayo score of at least 30% from baseline. As endoscopy was not performed at week 8 in VARSITY, for the vedolizumab and adalimumab UC analyses, clinical response was defined as a reduction in partial Mayo ≥2 points and ≥25% from baseline, with a decrease in rectal bleeding subscore ≥1 or absolute rectal bleeding subscore ≤1. For the CD analysis, clinical response was defined as a reduction in CDAI of at least 100 points from baseline. All outcomes were assessed at week 8. For UNIFI, as endoscopy was also assessed post-induction, the predictive value of eosinophil counts was also assessed for endoscopic improvement, defined as a Mayo endoscopic subscore <2. Secondary outcomes included the evaluation of trends in other hematologic parameters, including neutrophils, red blood cells, white blood cells, hemoglobin, monocyte, and lymphocyte count, and their association with clinical response.

### 2.3. Statistical analysis

Baseline characteristics were summarized using descriptive statistics for the study population. Dichotomous variables were expressed as proportions or percentages, while continuous variables were reported as means with standard deviations (SD) or medians with interquartile ranges (IQR). Categorical variables were compared using Chi-squared tests. To compare the sensitivity and specificity of the percent change in eosinophils from baseline on outcomes, receiver operating characteristic curve analyses were performed and evaluated using the area under the curve (AUC) defined as poor 0.5-0.7; moderate 0.7-0.8; good 0.8-0.9; and excellent 0.9-1.0.

Comparisons of eosinophil counts between responders and non-responders were performed at each time point using *t*-tests to determine statistical significance. Changes in eosinophil counts from baseline to week 8 were analyzed both in absolute terms and as percentage changes. Sensitivity analyses were planned among those using baseline corticosteroids and those not for the largest dataset available (UNIFI). In addition, logistic regression was used to evaluate the odds ratio (OR) and 95% confidence intervals (CI) for achieving clinical response based on elevated eosinophil counts at baseline. Lines of best fit were used to illustrate trends and relationships identified from logistic regression models. To evaluate the average percent change, the least squares mean was calculated for each comparison.

## 3. Results

Table 1 demonstrates the baseline characteristics of the study population. Age, disease duration, as well as corticosteroid and immunomodulator use were similar between groups. No differences were observed for baseline eosinophil, white blood cell, neutrophil, monocyte, or lymphocyte counts.

**Table 1.** Baseline characteristics of the study population.

Variable	UNIFI (Ustekinumab UC) (*n* = 582)	SEAVUE (Ustekinumab CD) (*n* = 191)	SEAVUE (Adalimumab CD) (*n* = 195)	VARSITY (Adalimumab UC) (*n* = 374)	VARSITY (Vedolizumab UC) (*n* = 375)	*P* value
Age, mean (SD)	41.2 (13.7)	41.1 (14.3)	42.5 (13.1)	42.2 (12.5)	41.8 (13.0)	.748
Female, *n* (%)	239 (41.1)	76 (40.0)	82 (42.1)	161 (43.0)	153 (40.8)	.542
Disease duration in years, mean (SD)	8.2 (6.8)	7.8 (5.3)	7.7 (4.4)	6.4 (6.0)	7.2 (7.2)	.363
Baseline eosinophil count (×10^9^/L), mean (SD)	0.20 (0.2)	0.16 (0.1)	0.14 (0.1)	0.19 (0.3)	0.20 (0.3)	.492
Corticosteroid use at baseline, *n* (%)	308 (53.0)	67 (35.1)	60 (30.8)	134 (35.8)	130 (34.7)	.102
Immunomodulator use at baseline, *n* (%)	166 (28.5)	48 (25.1)	58 (29.7)	98 (26.2)	99 (26.4)	.499
Baseline white blood cell count (×10^9^/L), mean (SD)	6.2 (5.4)	8.8 (5.6)	7.4 (5.7)	7.9 (5.6)	8.2 (6.3)	.274
Baseline neutrophil count (×10^9^/L), mean (SD)	2775.1 (1155.6)	2684.7 (974.7)	2550.8 (1002.6)	2615.5 (878.3)	2602 (946.0)	.743
Baseline monocyte count (×10^9^/L), mean (SD)	0.5 (0.3)	0.5 (0.3)	0.4 (0.4)	0.6 (0.3)	0.6 (0.4)	.376
Baseline lymphocyte count (×10^9^/L), mean (SD)	5.3 (2.1)	3.8 (4.3)	5.2 (4.6)	5.4 (5.3)	5.8 (5.5)	.209

### 3.1. Eosinophil trends in patients on ustekinumab for UC during induction

**Figure 1.**
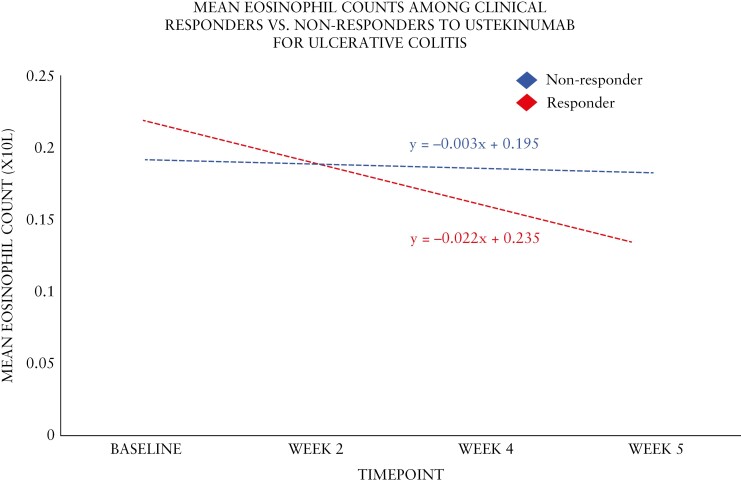
Absolute blood eosinophil count trendlines in patients on ustekinumab for ulcerative colitis during induction.

Among 582 patients treated with ustekinumab in the UNIFI study, eosinophil counts were assessed at various time points during the induction period (Table 2 and Figure 1). A total of 215/582 (36.9%) of participants were clinical responders and 175/582 (30.1%) achieved endoscopic improvement at week 8. At baseline (Week 0), the mean eosinophil count was significantly higher in week 8 clinical responders (0.21 × 10^9^/L, SD 0.2) compared to non-responders (0.18 × 10^9^/L, SD 0.2, *P* = .042). By week 8, clinical responders showed a mean eosinophil count of 0.14 × 10^9^/L (SD 0.1), which was lower than that of non-responders at 0.17 × 10^9^/L (SD 0.2). The absolute delta from baseline to week 8 was significantly different between responders and non-responders (−0.07 × 10^9^/L vs −0.01 × 10^9^/L, *P* < .001), as was the percent change (−33.33% vs −5.55%, *P* = .027) and demonstrated moderate predictive ability [AUC 0.71 (95% CI: 0.65-0.77)]. The least square mean difference at week 8 was 21% and 10% among clinical responders and non-responders, respectively. Similar declines in week 8 eosinophil levels were observed among week 52 clinical responders (−15% vs 0%, *P* = .007) and among patients who achieved week 52 endoscopic improvement (−40% vs −15.8%, *P* = .035) (Table S1). No differences were observed among participants treated with fixed dose versus weight-based dose of ustekinumab. Sensitivity analysis defining clinical response using the partial Mayo score (partial Mayo ≥2 points and ≥25% from baseline, with a decrease in rectal bleeding subscore ≥1 or absolute rectal bleeding subscore ≤1) demonstrated similar findings (Table S3). Similar trends were observed for endoscopic improvement, with significant differences in week 8 eosinophil counts and changes from baseline (*P* = .002 for absolute change and *P* = .043 for percent change). Percent change demonstrated moderate predictive ability for the outcome of week 8 clinical response [AUC 0.70 (95% CI: 0.63-0.78)]. Patients with UC treated with ustekinumab with an eosinophil level increase >10% by week 8 from baseline were less likely to respond by week 8 compared to patients with an increase <10% or no increase at all [OR: 0.67 (95% CI: 0.35-0.98), *P* = .042]. In addition, patients with an eosinophil decline >10% by week 8 from baseline were significantly more likely to respond by week 8 compared to those who did not have a decline [OR: 1.34 (95% CI: 1.01-1.75), *P* = .047].

**Table 2.** Absolute Blood eosinophil count trends in patients on ustekinumab for ulcerative colitis during induction (*n* = 582).

Mean eosinophil count, ×10^9^/L (SD)	Week 8 clinical response^a^			Week 8 endoscopic improvement^b^		
	Non-responders (*n* = 367)	Responders (*n* = 215)	*P* value	Achieved (*n* = 175)	Not achieved (*n* = 407)	*P* value
Week 0	0.18 (0.2)	0.21 (0.2)	.042	0.21 (0.2)	0.19 (0.2)	.339
Week 2	0.20 (0.2)	0.19 (0.2)	.659	0.18 (0.2)	0.20 (0.2)	.141
Week 4	0.20 (0.2)	0.18 (0.1)	.152	0.16 (0.1)	0.19 (0.2)	.042
Week 8	0.17 (0.2)	0.14 (0.1)	.053	0.12 (0.1)	0.16 (0.2)	.002
Week 8 absolute delta from baseline	−0.01 (0.2)	−0.07 (0.2)	<.001	−0.09 (0.2)	−0.03 (0.2)	<.001
Week 8 percent delta from baseline	−5.55% (14.1)	−33.33% (10.7)	.027	−42.86% (15.4)	−15.79% (14.2)	.043

^a^Reduction in total Mayo score of at least 30% from baseline.

^b^Mayo endoscopic subscore < 2.

Sensitivity analyses were performed to assess the effect of corticosteroid use on outcomes (Tables S4 and S5). In the subgroup of patients not using corticosteroids at baseline, baseline eosinophil counts did not differ significantly between week 8 clinical responders and non-responders (0.23 × 10^9^/L vs 0.20 × 10^9^/L, *P* = .117). However, by week 8, responders had lower eosinophil counts compared to non-responders (0.14 × 10^9^/L vs 0.18 × 10^9^/L). The absolute and percent changes from baseline to week 8 were significantly different (*P* < .001 for both). Percent change from baseline demonstrated moderate predictive ability for week 8 clinical response [AUC 0.71 (95% CI: 0.65-0.78)] and week 8 endoscopic improvement [AUC 0.70 (95% CI: 0.63-0.75)]. The least square mean difference at week 8 was 18% and 5% among responders and non-responders, respectively. Endoscopic improvement showed similar patterns, with significant differences in week 8 eosinophil counts and changes from baseline (*P* = .021 for absolute delta and *P* < .001 for percent delta). Among patients using corticosteroids at baseline, week 8 clinical responders and non-responders had similar baseline eosinophil counts (0.21 × 10^9^/L vs 0.18 × 10^9^/L, *P* = .325). By week 8, responders had lower eosinophil counts compared to non-responders (0.15 × 10^9^/L vs 0.19 × 10^9^/L), and again the absolute and percent changes from baseline to week 8 were significant (*P* < .001 for both). Percent change from baseline demonstrated moderate predictive ability for the outcomes of week 8 clinical response [AUC 0.71 (95% CI: 0.65-0.78)] and week 8 endoscopic improvement [AUC 0.70 (95% CI: 0.63-0.75)]. Patients who attained week 8 endoscopic improvement were also more likely to demonstrate decreases in eosinophil counts and changes from baseline compared to those who did not attain endoscopic improvement (*P* = .047 for absolute delta and *P* < .001 for percent delta). The least square mean difference at week 8 was 21% and 9% among responders and non-responders, respectively.

### 3.2. Eosinophil trends in patients on ustekinumab for Crohn’s disease during induction

**Figure 2.**
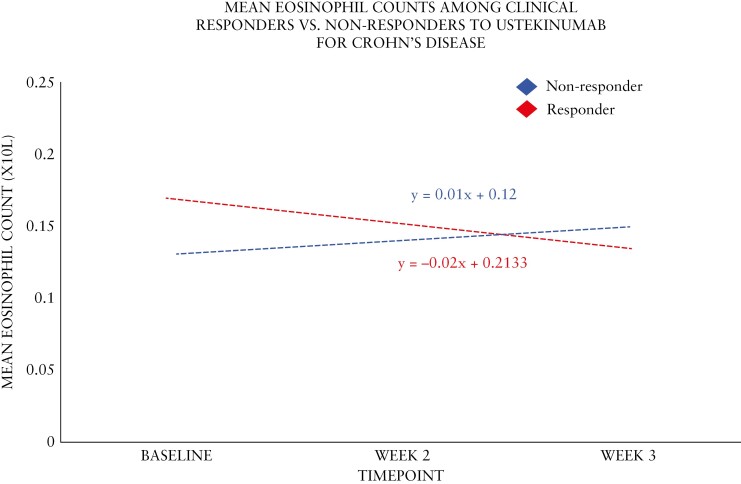
Absolute blood eosinophil count trendlines in patients on ustekinumab for Crohn’s disease during induction.

Blood eosinophil count trends were assessed among 191 participants with CD from SEAVUE who were treated with ustekinumab. Week 8 clinical responders had a significantly higher baseline eosinophil count compared to non-responders (0.18 × 10^9^/L vs 0.12 × 10^9^/L, *P* = .003) (Table 3 and Figure 2). However, by week 8, there was no significant difference (0.14 × 10^9^/L for both), and the absolute and percent changes from baseline to week 8 were significantly different in responders as compared to non-responders (*P* = .024 and *P* = .004, respectively), with percent change from baseline demonstrating moderate predictive ability for week 8 clinical response [AUC 0.72 (95% CI: 0.64-0.79)]. The least square mean difference at week 8 was 14% and 8% among responders and non-responders, respectively. Similar declines in week 8 eosinophil levels were also observed among week 52 clinical responders (Table S2). Similar to ustekinumab patients with UC, those with CD treated with ustekinumab who had an eosinophil level increase >10% from baseline were also less likely to respond by week 8 compared to patients with an increase <10% or no increase at all [OR: 0.71 (95% CI: 0.41-0.94), *P* = .048]. Compared to those with no decline in eosinophil levels from baseline, those with a decline >10% by week 8 from baseline were significantly more likely to achieve clinical response at week 8 [OR: 1.32 (95% CI: 0.99-1.79), *P* = .042].

**Table 3.** Absolute Blood eosinophil count trends in patients with Crohn’s disease on ustekinumab during induction (*n* = 191).

Mean eosinophil count, ×10^9^/L (SD)	Week 8 clinical response^a^		
	Non-responders	Responders	*P*-value
Week 0	0.12 (0.1)	0.18 (0.1)	.003
Week 2	0.16 (0.1)	0.20 (0.2)	.047
Week 8	0.14 (0.1)	0.14 (0.1)	.523
Week 8 absolute delta from baseline	0.02 (0.1)	−0.04 (0.1)	.024
Week 8 percent delta from baseline	16.67% (66.6)	−22.22% (69.6)	.004

^a^Reduction in CDAI of at least 100 points from baseline.

### 3.3. Blood eosinophil count trends in patients on adalimumab for UC during induction

**Figure 3.**
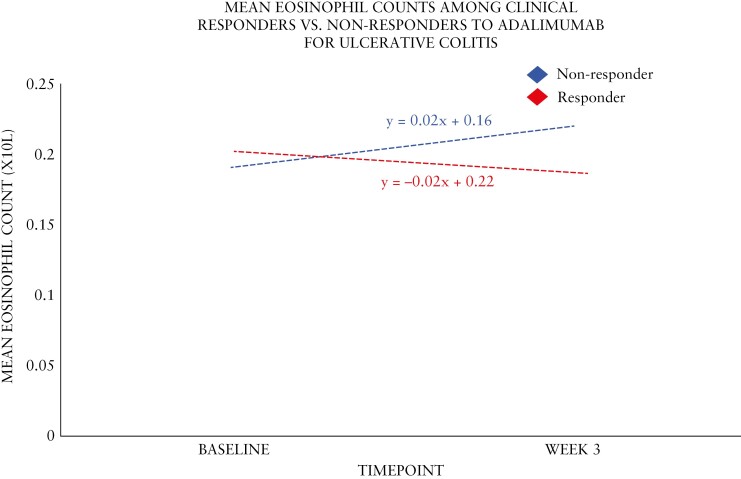
Absolute blood eosinophil count trendlines in patients on adalimumab for ulcerative colitis during induction.

Among 374 participants with UC treated with adalimumab, week 8 clinical responders had similar baseline eosinophil counts compared to week 8 non-responders (0.20 × 10^9^/L vs 0.19 × 10^9^/L, *P* = .839) (Table 4 and Figure 3). Eosinophil counts declined among clinical responders and increased in non-responders by week 6, although this was not significantly different. The least square mean difference at week 6 was 12% and 9% among responders and non-responders, respectively.

**Table 4.** Absolute blood eosinophil count trends in patients on adalimumab for ulcerative colitis during induction (*n* = 374).

Mean eosinophil count, ×10^9^/L (SD)	Week 8 clinical response^a^		
	Non-responders	Responders	*P* value
Week 0	0.19 (0.27)	0.20 (0.24)	.839
Week 6	0.22 (0.25)	0.18 (0.20)	.082
Week 6 absolute delta from baseline	0.03 (0.02)	−0.02 (0.04)	.711
Week 6 percent delta from baseline	15.8 (65.2)	10.0 (60.0)	.247

^a^Response defined as partial Mayo ≥ 2 points and ≥25% from baseline, with a decrease in rectal bleeding subscore ≥ 1 or absolute rectal bleeding subscore ≤ 1.

^b^Mayo endoscopic subscore < 2.

### 3.4. Blood eosinophil count trends in patients on adalimumab for Crohn’s disease during induction

**Figure 4.**
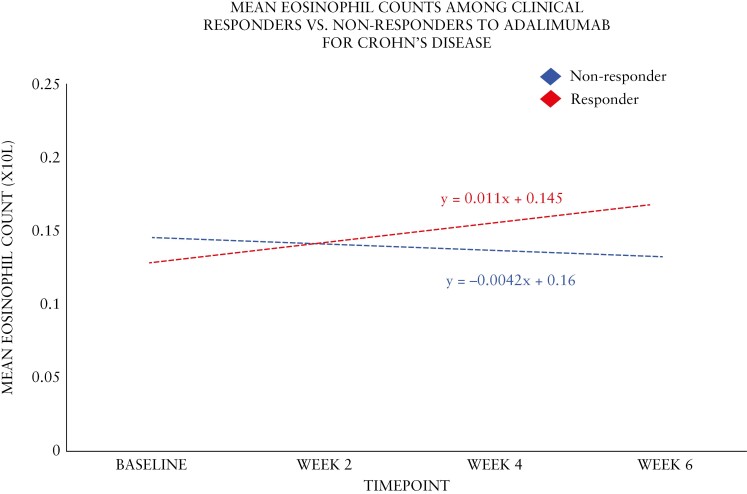
Absolute blood eosinophil count trendlines in patients on adalimumab for Crohn’s disease during induction.

When 195 patients with CD who were treated with adalimumab were assessed, there were no significant differences in eosinophil counts between Week 8 clinical responders and non-responders at any time point (baseline, Week 2, and Week 8). Changes from baseline to Week 8 in both absolute and percent terms were also not significantly different (Table 5 and Figure 4). The least square mean difference at week 8 was 3% among responders and non-responders.

**Table 5.** Absolute blood eosinophil count trends in patients on adalimumab for Crohn’s disease during induction (*n* = 195).

Mean eosinophil count, ×10^9^/L (SD)	Week 8 clinical response^a^		
	Non-responders	Responders	*P* value
Week 0	0.14 (0.1)	0.14 (0.1)	.706
Week 2	0.15 (0.1)	0.16 (0.1)	.761
Week 4	N/A	N/A	N/A
Week 8	0.13 (0.1)	0.15 (0.1)	.401
Week 8 absolute delta from baseline	0.01 (0.1)	0.01 (0.1)	.248
Week 8 percent delta from baseline	−7.14% (39.5)	−7.14% (39.5)	.245

^a^Reduction in CDAI of at least 100 points from baseline.

### 3.5. Eosinophil trends in patients on vedolizumab for UC during induction

Sensitivity analyses were also performed among 375 vedolizumab-treated patients with UC from the VARSITY study with available data (Table S6). Baseline eosinophil counts were similar among week 8 clinical responders and non-responders (0.18 × 10^9^/L vs 0.19 × 10^9^/L, *P* = .742). Similar findings were observed at week 6 (0.20 × 10^9^/L vs 0.21 × 10^9^/L, *P* = .426). The least square mean difference at week 8 was 5% among responders and 3% among non-responders.

### 3.6. Post-induction clinical response stratified by baseline eosinophil levels

To determine whether baseline eosinophilia served as a marker to predict treatment response, Table 6 compares clinical response rates among participants who were treated with adalimumab or ustekinumab stratified by the presence of elevated serum eosinophils at baseline. No significant differences were observed among patients with CD or UC.

**Table 6.** Comparison of post-induction clinical response rates stratified by baseline absolute blood eosinophil counts.

	Elevated eosinophils at baseline	Not elevated eosinophils at baseline	OR (95% CI)	*P* value
UNIFI (ustekinumab) *ULN eosinophils is 0.57*	19/27 (70.4)	342/555 (61.6)	0.72 (0.40-1.29)	.360
SEAVUE (ustekinumab) *ULN eosinophils is 0.57*	17/22 (77.3)	136/169 (80.5)	0.91 (0.35-2.39)	.724
SEAVUE (adalimumab) *ULN eosinophils is 0.57*	12/14 (85.7)	150/181 (82.9)	1.11 (0.33-3.74)	.785
VARSITY (adalimumab) *ULN eosinophils is 0.80*	4/5 (80.0)	183/369 (49.6)	4.07 (0.45-36.72)	.177

### 3.7. Trends in other hematologic parameters

Tables S7–12 present various hematologic parameters in patients treated with ustekinumab for UC and CD, stratified by clinical response at week 8. For the mean absolute neutrophil count, white blood cell count, red blood cell count, hemoglobin count, and monocyte count, there were no significant differences between responders and non-responders at baseline, week 8, or in the changes from baseline to week 8. A trend was observed in the change in lymphocyte count from baseline to week 8.

## 4. Discussion

Recent studies have evaluated blood eosinophil counts as potential biomarkers for autoimmune diseases such as IBD.^9^ Elevated eosinophil counts and increased eosinophil activation have been associated with active disease states in IBD. Monitoring changes in eosinophil levels in response to biological therapies could provide valuable insights into the effectiveness of treatment. For instance, a rapid decline in eosinophil counts following the initiation of ustekinumab therapy might indicate a positive therapeutic response, while a rise in eosinophil counts could predict the need for alternative treatment approaches. In our study, we identified a reduction in blood eosinophil count by week 8 among responders to ustekinumab in both UC and CD, but not adalimumab for UC and CD or vedolizumab for UC.

In our study, we identified the utility of blood eosinophil count decline as a marker for early response to ustekinumab in CD and UC, but not adalimumab or vedolizumab. We also demonstrated that change in eosinophil levels by week 8 is a robust marker for predicting long-term outcomes at week 52. One hypothesis as to why this was observed is while eosinophils produce TNF-alpha, anti-TNFs such as adalimumab block TNF-alpha but do not act further upstream.^10,11^ On the contrary, IL-23 has been linked to eosinophil production.^12^ This may suggest that targeting the IL-23 pathway may be more effective in managing eosinophil-associated inflammation in IBD compared to targeting TNF-alpha alone. As eosinophils express receptors for IL-12 and IL-23, blockade of IL-12 and IL-23 using ustekinumab may be effective in reducing eosinophil levels, which was observed among responders in our study.^13^ Monitoring eosinophil counts during induction therapy with ustekinumab could offer clinicians an early indication of treatment efficacy, potentially guiding therapeutic decisions and optimizing patient outcomes. We observed that participants with UC and CD who had a >10% increase in eosinophil levels from baseline were less likely to achieve clinical response at week 8, which further strengthens the hypothesis that eosinophil levels can help predict treatment response. Moreover, our study underscores the importance of biomarker research in personalized medicine approaches for inflammatory bowel diseases, paving the way for future investigations into the mechanistic roles of eosinophils in treatment response pathways.

Beyond eosinophils, we assessed various hematologic parameters to identify whether the effect observed in patients treated with ustekinumab was observed only with eosinophils. Although there were some numerical differences observed, such as a greater reduction in neutrophil and red blood cell counts in week 8 clinical responders, none of these reached statistical significance. These findings suggest that these hematologic parameters did not predict clinical response to ustekinumab, and provide further confidence that markers such as eosinophils may be an independent biomarker that predicts treatment efficacy.

The role of eosinophils in the inflammatory cascade of IBD underscores their potential as targets for novel therapeutic interventions. In the context of ustekinumab treatment, changes in eosinophil counts may reflect changes in the inflammatory state of the gastrointestinal tract.^14^ High baseline eosinophil counts could indicate ongoing inflammation and disease activity, whereas a reduction in eosinophil numbers over time may suggest a therapeutic response to ustekinumab-induced cytokine inhibition and decreased inflammation. This hypothesis is supported by clinical observations showing that patients who achieve early clinical response to ustekinumab experienced reductions in eosinophil counts.

By understanding the mechanisms of eosinophil recruitment and activation in the gastrointestinal tract, new strategies can be developed to modulate their activity and mitigate inflammation. This could lead to more precise targeting of the inflammatory pathways involved in IBD, ultimately improving patient outcomes. As research continues to elucidate the complex interactions between eosinophils and other immune cells in IBD, the potential for eosinophil-based biomarkers and therapies becomes increasingly promising. Blockade of IL-4 and IL-13 using dupilumab is currently being assessed in a phase 2 study for patients with UC (NCT05731128), and the results may provide valuable insights into its efficacy and potential as a treatment option. Future studies should focus on validating eosinophil-related markers in large heterogenous populations and exploring the therapeutic benefits of targeting eosinophil pathways in combination with existing biological treatments.

Strengths of our study include the use of robust participant-level data from multiple phase 3 and 4 clinical trials. However, several limitations are worth noting. First, we were unable to assess the relationship between eosinophil improvement and endoscopic improvement among participants with CD, as endoscopy was not routinely performed at post-induction in these trials. Second, our statistical analyses may overlook adjustments for multiple comparisons, potentially increasing the risk of false positives. In addition, the study’s focus on eosinophil counts as biomarkers overlooks other potentially influential factors such as concomitant medications and disease severity, which could confound the observed associations. Notably, we performed subgroup analyses on participants stratified by steroid use and observed that trends in eosinophil count reduction were similar among responders and non-responders regardless of steroid use. Furthermore, prior exposure to biologics can potentially influence treatment response, especially with regard to immune modulation pathways. However, we were unable to assess the impact of prior biologic treatment on response in our study. Findings from this study should be interpreted as hypothesis-generating and not confirmatory. Further studies with additional datasets are warranted to better understand our findings.

In conclusion, while our findings indicate that other hematologic parameters did not predict clinical response to ustekinumab, the significant differences observed in eosinophil counts and their changes from baseline suggest that eosinophils may serve as a more relevant marker for assessing efficacy of ustekinumab among patients with CD and UC. This further supports the need for ongoing research into eosinophil-based biomarkers and therapies to enhance the management of IBD.

## Supplementary Material

jjaf006_suppl_Supplementary_Tables

## Data Availability

This publication (Vivli protocol #00007656) is based on research using data from Janssen Inc. and Takeda Inc. that has been made available through Vivli, Inc. Vivli has not contributed to or approved, and is not in any way responsible for, the contents of this publication.
