# Supplementary material for: Improvement in serum eosinophilia is observed in clinical responders to ustekinumab but not adalimumab in inflammatory bowel disease
Source: J Crohns Colitis. 2025 Jan 13;19(1):jjaf006. doi: 10.1093/ecco-jcc/jjaf006 (PMC11760988; doi:10.1093/ecco-jcc/jjaf006)
Supplement: jjaf006_suppl_Supplementary_Tables [file jjaf006_suppl_supplementary_tables.docx]

| Mean eosinophil count, x10^9^/L (SD) | Week 8 Clinical Response* | | |
| --- | --- | --- | --- |
|  | Non-responders (n=332) | Responders (n=250) | p-value |
| Week 0 | 0.17 (0.2) | 0.21 (0.2) | 0.037 |
| Week 2 | 0.21 (0.2) | 0.19 (0.2) | 0.745 |
| Week 4 | 0.21 (0.2) | 0.17 (0.1) | 0.252 |
| Week 8 | 0.16 (0.2) | 0.14 (0.1) | 0.099 |
| Week 8 absolute delta from baseline | -0.01 (0.2) | -0.07 (0.2) | <0.001 |
| Week 8 percent delta from baseline | -5.55% (14.1) | -33.33% (10.7) | 0.004 |

Supplementary Table 1 - Absolute Blood eosinophil count trends in patients on ustekinumab for ulcerative colitis during induction

*Reduction in total Mayo score of at least 30% from baseline
**Mayo endoscopic subscore < 2

| Mean eosinophil count, x10^9^/L (SD) | Week 52 Clinical Response* | | | Week 52 Endoscopic Improvement** | | |
| --- | --- | --- | --- | --- | --- | --- |
|  | Non-responders (n=181) | Responders (n=401) | p-value | Achieved (n=320) | Not achieved (n=262) | p-value |
| Week 0 | 0.18 (0.2) | 0.20 (0.2) | 0.037 | 0.20 (0.2) | 0.19 (0.2) | 0.294 |
| Week 2 | 0.21 (0.2) | 0.19 (0.2) | 0.645 | 0.18 (0.2) | 0.21 (0.2) | 0.253 |
| Week 4 | 0.20 (0.2) | 0.19 (0.1) | 0.342 | 0.15 (0.1) | 0.19 (0.2) | 0.038 |
| Week 8 | 0.18 (0.2) | 0.17 (0.1) | 0.104 | 0.12 (0.1) | 0.16 (0.2) | 0.002 |
| Week 8 absolute delta from baseline | 0 (0.2) | -0.03 (0.1) | 0.029 | -0.08 (0.1) | -0.03 (0.1) | 0.042 |
| Week 8 percent delta from baseline | 0% | -15% (14.2) | 0.007 | -40% (15.7) | -15.8% (16.3) | 0.035 |

Supplementary Table 2 – Absolute Blood eosinophil count trends in patients with Crohn’s disease on ustekinumab during induction on week 52 outcomes

| Mean eosinophil count, x10^9^/L (SD) | Week 52 Clinical Response* | | |
| --- | --- | --- | --- |
|  | Non-responders | Responders | p-value |
| Week 0 | 0.14 (0.1) | 0.18 (0.1) | 0.037 |
| Week 2 | 0.14 (0.1) | 0.21 (0.1) | 0.002 |
| Week 8 | 0.17 (0.1) | 0.13 (0.1) | 0.007 |
| Week 8 absolute delta from baseline | 0.03 (0.1) | -0.05 (0.1) | 0.001 |
| Week 8 percent delta from baseline | 21.4% (43.6) | -27.7% (37.9) | 0.001 |

*Reduction in CDAI of at least 100 points from baseline

Supplementary Table 3 – Sensitivity analysis using partial Mayo score reduction in absolute blood eosinophil count trends in patients on ustekinumab for ulcerative colitis during induction (n=582)

*Response defined as partial Mayo ≥2 points and ≥25% from baseline, with a decrease in rectal bleeding subscore ≥1 or absolute rectal bleeding subscore ≤1

Supplementary Table 3 – Absolute blood eosinophil counts in patients on ustekinumab for ulcerative colitis during induction without corticosteroid use at baseline (n=373)

| Mean eosinophil count, x10^9^/L (SD) | Week 8 Clinical Response* | | | Week 8 Endoscopic Improvement** | | |
| --- | --- | --- | --- | --- | --- | --- |
|  | Non-responders | Responders | p-value | Achieved | Not achieved | p-value |
| Week 0 | 0.20 (0.2) | 0.23 (0.2) | 0.117 | 0.23 (0.2) | 0.21 (0.2) | 0.277 |
| Week 2 | 0.22 (0.2) | 0.20 (0.2) | 0.441 | 0.19 (0.2) | 0.21 (0.2) | 0.278 |
| Week 4 | 0.22 (0.3) | 0.19 (0.1) | 0.066 | 0.18 (0.1) | 0.21 (0.2) | 0.210 |
| Week 8 | 0.18 (0.2) | 0.14 (0.1) | 0.017 | 0.12 (0.1) | 0.16 (0.2) | 0.021 |
| Week 8 absolute delta from baseline | -0.02 (0.2) | -0.09 (0.2) | <0.001 | -0.11 (0.2) | -0.05 (0.2) | 0.002 |
| Week 8 percent delta from baseline | -10.53% (17.8) | -39.13% (56.4) | <0.001 | -47.83% (35.4) | -19.05% (43.4) | <0.001 |

*Reduction in total Mayo score of at least 30% from baseline
**Mayo endoscopic subscore < 2

Supplementary Table 4 – Absolute blood eosinophil counts in patients on ustekinumab for ulcerative colitis during induction with corticosteroid use at baseline (n=209)

| Mean eosinophil count, x10^9^/L (SD) | Week 8 Clinical Response* | | | Week 8 Endoscopic Improvement** | | |
| --- | --- | --- | --- | --- | --- | --- |
|  | Non-responders | Responders | p-value | Achieved | Not achieved | p-value |
| Week 0 | 0.18 (0.2) | 0.21 (0.2) | 0.325 | 0.22 (0.1) | 0.21 (0.2) | 0.452 |
| Week 2 | 0.19 (0.3) | 0.21 (0.2) | 0.423 | 0.18 (0.2) | 0.21 (0.2) | 0.524 |
| Week 4 | 0.21 (0.2) | 0.20 (0.2) | 0.734 | 0.17 (0.1) | 0.21 (0.2) | 0.111 |
| Week 8 | 0.19 (0.2) | 0.15 (0.1) | 0.011 | 0.12 (0.1) | 0.15 (0.2) | 0.047 |
| Week 8 absolute delta from baseline | 0.01 (0.2) | -0.06 (0.2) | <0.001 | -0.10 (0.2) | -0.07 (0.2) | 0.031 |
| Week 8 percent delta from baseline | -5.55% (12.5) | -28.57% (23.5) | <0.001 | -45.45% (22.6) | -33.33% (23.4) | <0.001 |

*Reduction in total Mayo score of at least 30% from baseline
**Mayo endoscopic subscore < 2

Supplementary Table 5 - Absolute blood eosinophil count trends in patients on vedolizumab for ulcerative colitis during induction (n=375)

| Mean eosinophil count, x10^9^/L (SD) | Week 8 Clinical Response* | | |
| --- | --- | --- | --- |
|  | Non-responders | Responders | p-value |
| Week 0 | 0.18 (0.16) | 0.19 (0.20) | 0.742 |
| Week 6 | 0.20 (0.23) | 0.21 (0.22) | 0.426 |
| Week 6 absolute delta from baseline | 0.02 (0.07) | 0.02 (0.02) | 0.341 |
| Week 6 percent delta from baseline | 11.1 (43.8) | 10.5 (10.0) | 0.121 |

* Response defined as partial Mayo ≥2 points and ≥25% from baseline, with a decrease in rectal bleeding subscore ≥1 or absolute rectal bleeding subscore ≤1

**Mayo endoscopic subscore < 2

Supplementary Table 6 – Absolute blood neutrophil count in patients with UC and CD on ustekinumab

| Mean absolute neutrophil count, (SD) | Week 8 Clinical Response* | | |
| --- | --- | --- | --- |
|  | Non-responders | Responders | p-value |
| Week 0 | 2605.8 (1005.2) | 2845.7 (1103.4) | 0.523 |
| Week 2 | 2763.7 (1205.6) | 2664.8 (1006.2) | 0.426 |
| Week 4 | 2687.2 (1114.5) | 2653.4 (1529.6) | 0.886 |
| Week 8 | 2567.2 (1256.7) | 2776.2 (1004.2) | 0.437 |
| Week 8 absolute delta from baseline | -38.6 (251.5) | -69.5 (99.2) | 0.337 |
| Week 8 percent delta from baseline | -1.48% (20.5) | -2.44% (11.5) | 0.246 |

Supplementary Table 7 – Absolute blood white blood cell count in patients with UC and CD on ustekinumab

| Mean white blood cell count, x10^9^/L (SD) | Week 8 Clinical Response* | | |
| --- | --- | --- | --- |
|  | Non-responders | Responders | p-value |
| Week 0 | 6.3 (4.6) | 8.5 (5.9) | 0.252 |
| Week 2 | 6.5 (4.8) | 9.2 (3.6) | 0.452 |
| Week 4 | 7.4 (3.2) | 8.5 (6.0) | 0.354 |
| Week 8 | 6.8 (3.2) | 9.4 (5.8) | 0.643 |
| Week 8 absolute delta from baseline | 0.5 (1.4) | 0.9 (0.1) | 0.524 |
| Week 8 percent delta from baseline | 7.9% (3.2) | 10.6% (5.8) | 0.243 |

Supplementary Table 8 – Absolute blood red blood cell count in patients with UC and CD on ustekinumab

| Mean red blood cell count, x10^12^/L (SD) | Week 8 Clinical Response* | | |
| --- | --- | --- | --- |
|  | Non-responders | Responders | p-value |
| Week 0 | 6.4 (2.1) | 7.4 (3.5) | 0.425 |
| Week 2 | 6.2 (2.2) | 7.4 (3.7) | 0.589 |
| Week 4 | 6.8 (3.2) | 7.1 (3.7) | 0.639 |
| Week 8 | 6.2 (4.6) | 6.9 (4.1) | 0.799 |
| Week 8 absolute delta from baseline | -0.2 (2.5) | -0.5 (1.4) | 0.525 |
| Week 8 percent delta from baseline | -3.1% (0.4) | -6.5% (2.1) | 0.240 |

Supplementary Table 9 – Hemoglobin count in in patients with UC and CD on ustekinumab

| Mean hemoglobin count, g/L (SD) | Week 8 Clinical Response* | | |
| --- | --- | --- | --- |
|  | Non-responders | Responders | p-value |
| Week 0 | 116.2 (20.7) | 110.6 (30.7) | 0.527 |
| Week 2 | 126.2 (34.8) | 106.9 (20.7) | 0.265 |
| Week 4 | 125.7 (18.5) | 120.7 (20.7) | 0.367 |
| Week 8 | 125.9 (19.2) | 116.2 (27.5) | 0.173 |
| Week 8 absolute delta from baseline | 9.7 (1.5) | 5.6 (3.2) | 0.214 |
| Week 8 percent delta from baseline | 8.3% (12.5) | 5.1% (10.4) | 0.647 |

Supplementary Table 10 – Absolute blood monocyte count in patients with UC and CD on ustekinumab

| Mean monocyte count, (SD) | Week 8 Clinical Response* | | |
| --- | --- | --- | --- |
|  | Non-responders | Responders | p-value |
| Week 0 | 0.5 (0.3) | 0.5 (0.4) | 0.867 |
| Week 2 | 0.5 (0.3) | 0.6 (0.4) | 0.764 |
| Week 4 | 0.5 (0.2) | 0.6 (0.3) | 0.734 |
| Week 8 | 0.5 (0.2) | 0.6 (0.3) | 0.523 |
| Week 8 absolute delta from baseline | 0 | 0.1 (0.1) | 0.990 |
| Week 8 percent delta from baseline | 0 | 20% (21.2) | n/a |

Supplementary Table 11 – Absolute blood lymphocyte count in patients with UC and CD on ustekinumab

| Mean lymphocyte count, (SD) | Week 8 Clinical Response* | | |
| --- | --- | --- | --- |
|  | Non-responders | Responders | p-value |
| Week 0 | 3.5 (1.7) | 4.2 (1.9) | 0.735 |
| Week 2 | 3.2 (1.8) | 4.0 (2.1) | 0.214 |
| Week 4 | 3.3 (1.9) | 3.9 (2.5) | 0.286 |
| Week 8 | 3.3 (2.0) | 4.3 (1.9) | 0.119 |
| Week 8 absolute delta from baseline | -0.2 (0.3) | 0.1 (0.1) | 0.103 |
| Week 8 percent delta from baseline | -5.7% (1.4) | 2.4 (1.8) | 0.064 |
